# Comparative Effectiveness of the Deqi Sensation and Non-Deqi by Moxibustion Stimulation: A Multicenter Prospective Cohort Study in the Treatment of Knee Osteoarthritis

**DOI:** 10.1155/2013/906947

**Published:** 2013-08-21

**Authors:** Rixin Chen, Mingren Chen, Jun Xiong, Tongsheng Su, Meiqi Zhou, Jianhua Sun, Zhenhai Chi, Bo Zhang, Dingyi Xie

**Affiliations:** ^1^The Affiliated Hospital with Jiangxi University of TCM, Nanchang 330006, China; ^2^Shanxi TCM Hospital, Xian 710003, China; ^3^The First Affiliated Hospital with Anhui University of TCM, Hefei 230031, China; ^4^Jiangsu TCM Hospital, Nanjing 210029, China

## Abstract

Substantial evidence has supported that moxibustion stimulates a unique phenomenon of Deqi, heat-sensitive moxibustion sensation. This study consisted of a multicenter, prospective cohort study with two parallel arms (A: heat-sensitive moxibustion sensation group; B: nonheat-sensitive moxibustion sensation group). All forms of moxibustion were applied unilaterally on the right leg with a triangle shape of three acupuncture points simultaneously (bilateral Xi Yan (EX-LE5) and He Ding (EX-LE2)). After one month the primary outcome parameter GPCRND-KOA showed significant differences between groups: trial group 5.23 ± 2.65 (adjusted mean ± SE) 95% CI [4.44*~*6.01] versus control group 7.43 ± 2.80 [6.59*~*8.26], *P* = 0.0001. Significant differences were manifested in total M-JOA score during the follow-up period (*P* = 0.0006). Mean knee circumference indicated significant difference between the groups (*P* = 0.03; *P* = 0.007). Overall, this evidence suggested that the effectiveness of the Deqi sensation group might be more superior than the non-Deqi sensation one in the treatment of KOA. This study was aimed at providing scientific evidence on the Deqi sensation of moxibustion and at showing that heat-sensitive moxibustion sensation is essential to achieve the preferable treatment effects of KOA.

## 1. Background

Acupuncture stimulates the Deqi, a sensory response which literally means “the arrival of meridian Qi” according to traditional Chinese medicine (TCM) [1, 2]. The classical TCM textbook of *Huangdi Neijing* states that the Deqi must be felt by the therapist, and it is also necessary for therapist to concentrate in order to ensure the Deqi [3]. The essence of acupuncture therapy was expressed in *Nine Needle* and *Twelve Sources* (from *Huangdi Neijing*), “the arrival of meridian Qi ensures the therapeutic effects.” This chapter describes the importance of activating meridian Qi and prompting it to transmit to the affected body part. Therefore, a lot of researchers confirmed that the Deqi is the key experience related to clinical efficacy of acupuncture [4, 5].

For acupuncture needle, multiple unique sensations experienced by the patient around the applied part of needle manipulation are often described as suan (aching or soreness), ma (numbness or tingling), zhang (fullness/distention or pressure), and zhong (heaviness) [6]. The Deqi stimulated by needle is believed to be closely related to clinical effects [7]. And there is also evidence to support that the increasing clinical effects were associated with the Deqi by needle stimulation [8–10].

Unlike acupuncture needle, which involves thrusting or twisting of needles and induces various Deqi phenomena, moxibustion implements heat stimulation of various temperature levels from mild skin warming to acupuncture points. Suspended moxibustion is the most common therapy in China. It involves burning of moxa on the acupuncture points at a distance. The Deqi by moxibustion stimulation is different from the one simulated by acupuncture needle as well. Substantial evidence has supported that moxibustion stimulates a unique of Deqi, that is, heat-sensitive moxibustion sensation [11]. A lot of observations and researches were adopted to confirm this phenomenon in the 1990s [12].

For humans, acupuncture points include two states: stimulated state and resting state. For healthy people, acupuncture points are in a resting state, and moxibustion only stimulates local superficial heat sensation. When people get sick, the acupuncture points on the surface of body are activated and sensitized. Several Deqi sensations are induced and called heat-sensitive moxibustion sensation. The first of all, penetrating heat, is the feeling from the applied part of the skin sinking into the underlying tissues or organs. In the second, expanding heat is the feeling of heat spreading out from the spot receiving moxibustion. The third, transmitting heat, refers to the sensation of heat moving from spot receiving moxibustion along a certain route. These sensations indicate that meridian Qi has been stimulated, and transmission has occurred [13]. However, there is lack of experimental data to indicate how heat-sensitive moxibustion sensation (the Deqi by moxibustion stimulation) compares with conventional local superficial heat sensation (non-Deqi by moxibustion stimulation).

Moreover, several articles and research reports have reported the effectiveness and safety of moxibustion for the treatment of knee osteoarthritis (KOA) [14–17]. Moxibustion has anti-inflammatory or immunomodulatory effects to fight against chronic inflammatory conditions in humans. For KOA, especially, is it necessary for moxibustion to produce the phenomenon of obtaining Qi in order to improve the curative effect? Therefore, it would be valuable to know whether there is difference between the moxibustion sensations in the treatment of KOA. The rigorous multicenter prospective cohort study trial was planned in order to determine the relationship of the Deqi by moxibustion stimulation and therapeutic effect.

## 2. Methods

### 2.1. Objective

The aim of this study is to compare the effectiveness of heat-sensitive moxibustion sensation and nonheat-sensitive moxibustion sensation in the treatment of patients with moderate-to-severe swelling KOA in China.

### 2.2. Sample Size

The sample size for testing the difference between the effective rates was calculated by the SPSS 13.0 programme. The outcome was the guiding principle of clinical research on new drugs in the treatment of KOA (GPCRND-KOA) [18]. According to previous pilot study, the effective rate in heat-sensitive moxibustion sensation group is 80% and 50% in the other group. If we apply a two-sided 5% significance level, 90% power the calculated required sample size is approximately 36 participants in each group. Allowing for a 20% loss to followup, a total of 45 participants were required in two groups:

(1) $$\begin{array}{c} n={\left\{\frac{Z_{1-a}\sqrt{2pq}+Z_{1-\beta }\sqrt{p_{1}\left(1-p_{1}\right)+p_{2}\left(1-p_{2}\right)}}{p_{1}-p_{2}}\right\}}^{2},\\ p=\frac{\left(p_{1}+p_{2}\right)}{2},\;\;q=1-p. \end{array}$$

### 2.3. Design

A multicenter (four centers in China), prospective cohort study was conducted by the Affiliated Hospital of Jiangxi University of Traditional Chinese Medicine (TCM) in Nanchang, the first Affiliated Hospital of Anhui University of TCM in Hefei, Jiangsu TCM Hospital in Nanjing, and Shanxi TCM Hospital in Xian. The patients were recruited at either outpatient service or inpatient department and had already made their own choice of moxibustion therapy. Thus, the groups were divided by the appearance of acupuncture point's Deqi sensation stimulated by suspended moxibustion. In trial group, patients felt the Deqi sensation when the acupuncture point was stimulated by moxibustion heat. In the control group, patients felt local superficial heat sensation (non-Deqi sensation) when the acupuncture point was stimulated by moxibustion heat.

### 2.4. Participants

#### 2.4.1. Recruitment

Patients were recruited in China for this nonrandomized prospective multicenter open comparative cohort study from July 30, 2010 to July 30, 2011. The ethics committees of the Affiliated Hospital with Jiangxi University of TCM approved the study and the consent procedure. Oral and written informed consent was obtained after verbal information about the study was provided by the physician. The signed consent form was sent to the central study center, and a copy was kept at the physician's office.

#### 2.4.2. Inclusion Criteria

Participants meeting the following criteria were included: patients met the diagnostic criteria for GPCRND-KOA; the GPCRND-KOA scale for KOA count should be more than 5 points, moderate-to-severe swelling KOA; the age of patients was from at least 38 years to no more than 70 years, and regardless of genders. According to the following KOA diagnosis standard, the following criteria were included simultaneously: knee joints appeared swelling; floating patella test was negative; patients accepted the treatment protocol in this trial; patients had stopped receiving previous treatment before recruitment for two weeks.

#### 2.4.3. Exclusion Criteria

Participants with one or more than one of the following criteria should be excluded: participants suffered from serious life-threatening disease, such as the heart disease or disease of brain and blood vessels, liver, kidney, and hematopoietic system, as well as psychotic patients; patients with diabetes, diabetic polyneuropathy, and polyneuropathic disturbances; the pregnant patients or patients in lactation period. The following conditions were also excluded items: acute knee joint trauma or ulceration in its local skin, complicated with serious genu varus/valgus and flexion contraction.

### 2.5. Study Interventions

The moxibustion therapies were implemented by qualified specialists of acupuncture in TCM with at least five years of clinical experience in this study. All treatment regimens were standardized between four centers practitioners by means of video, hands-on training, and internet. Both groups of patients were requested to receive no other treatments such as physical therapies, pain-killing medicines, or acupuncture treatment from other places.

In the two groups, 22-millimeter (diameter) × 120-millimetre (length) moxa sticks (produced by Jiangxi provincial TCM Hospital, China) were applied. The patients usually laid in the comfortable supine position for treatment, with 24°C ~ 30°C temperature in the room. Loose trousers are suggested to wear, in order to make knee joints to be exposed.

#### 2.5.1. The Heat-Sensitive Moxibustion Sensation Group

The moxa sticks were ignited by the therapist, and three acupuncture points (bilateral Xi Yan (EX-LE5) and He Ding (EX-LE2)) with triangle shape should be implemented simultaneously by suspended moxibustion. The warm suspended moxibustion was applied 3 centimetres away from the surface of the skin to search for the heat-sensitive moxibustion sensation. In this group, these acupuncture points were brought mild warmth without burning by moxa sticks and manipulated until the patient reported the characteristic of heat sensitization sensation; it is commonly called Deqi. Patients felt comfortable in the moxibustion manipulation.

The following patients' sensation suggested the Deqi: penetrating heat sensation due to moxa heat, defining as the heat sensation conducting from the moxa local skin surface into deep tissue or even into the joint cavity; expanding heat sensation due to moxa heat, defining as the heat sensation spreading the surrounding little by little around the moxa point; transmitting heat sensation due to moxa heat, defining as the heat sensation transferring along some pathway or direction, even to the ankle or hip conduction. In the course of manipulation, the therapist continued for 15 minutes in per treatment session. Patients received the treatment two times/day in 1st week (one time/day from 2nd week) for a total of 35 sessions over 30 days.

#### 2.5.2. The Nonheat-Sensitive Moxibustion Sensation Group

Common practices were similar to the first group. Only one difference was that patients in this group felt local superficial heat sensation. No Deqi sensations were stimulated in this group.

### 2.6. Outcome Measures

Ministry of Health of the People's Republic of China (MHPRC) has proposed the GPCRND-KOA [18]. The GPCRND-KOA scale was used widely and authoritatively recommended by China Clinic Trial. In the scale, a patient with KOA was assessed, including pain, the relation between activity and pain, function impairment, and special exams (Table 1). This scoring system was previously validated [19]. The degree of KOA is divided into three levels: mild <5 scores; moderate 5~9 scores; severe >9 scores. In the terms of swelling knee, knee circumference was assessed at each time point. The parameter was measured in centimeters across the middle of a patella, with ordinary tape measure [20].

Therapeutic effect was evaluated by comparing baseline and final conditions reported by the patient. This trial also recorded adverse effects reported by patients during treatment. The outcome measures above were assessed before the treatment (month 0), at the end of the treatment period (month 1), and 6 months after the end of the treatment period (month 7).

### 2.7. Statistical Methods

Statistical analyses were based on the intention-to-treat (ITT) principle, including all patients with baseline values to receive treatment. All tests were exploratory and two sided with a level of significance of 5%. The statistician who conducted the analyses remained blind to treatment group, and data were only unblended once all data were summarized, and analyses were completed. Statistical analyses were performed according to a predefined statistical analysis plan using SAS for Windows, version 9.2 (SAS Institute, Cary, NC, USA). We adopted multilevel models analysis of covariance (ANCOVA) or generalized estimating equations (GEE). In these models, physicians considered random effects, and fixed effects were GPCRND-KOA (continuous), patient's age and gender, Body Mass Index (BMI), knee circumference (continuous), and previous treatment. Results are presented as adjusted mean or proportioned with a standard error (SE) and/or 95% confidence interval (CI).

### 2.8. Adverse Events

We defined adverse events as unfavorable or unintended signs, symptoms, or disease presenting after treatment; however, they were not necessarily related to the moxibustion intervention. Adverse events were analyzed descriptively by frequencies, percentages, and by chi-squared or Fisher's exact test (if feasible).

## 3. Results

### 3.1. Population and Baseline

**Figure 1:**
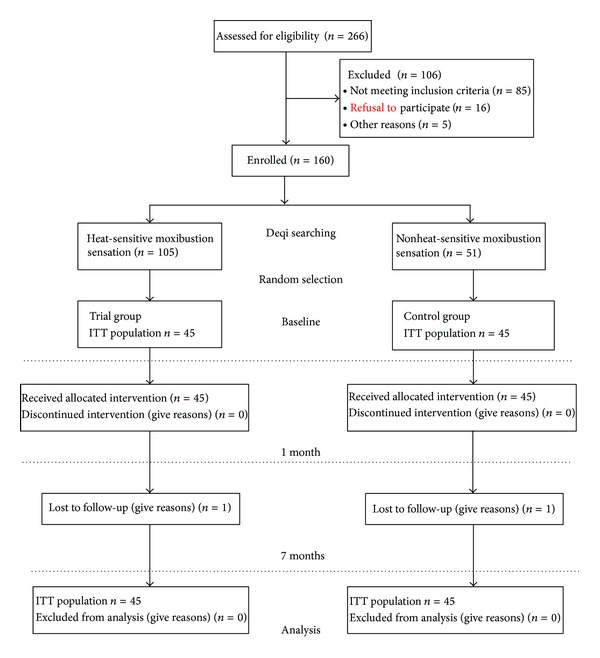
Flow diagram.

Among 266 screened patients, 106 could not be included in the study, mainly because they did not meet all eligibility criteria (Figure 1). Patients were recruited by 28 physicians experienced in the treatment of KOA (15 acupuncture doctors and 13 conventional doctors). After the search of the Deqi, 160 patients experienced heat-sensitive moxibustion sensation; 51 patients had no Deqi sensation. Since a sample of 90 people was calculated in our trial, we selected 45 patients from each queue separately by random drawing.

After seven months, 2 patients missed. Reasons for missing follow-up data were not contactable.

Patients' preferences resulted in the following baseline differences: patients in the trial group showed lower severe BMI scores, while the GPCRND-KOA score was higher in the control group. The males of the trial group were more than those of control group. In the previous treatment, there were obviously differences among the two treatment groups, as well as gender (Table 2). 

### 3.2. Outcome Parameters

#### 3.2.1. Total GPCRND-KOA Score

After 1 month the primary outcome parameter GPCRND-KOA showed significant differences between groups: trial group 5.23 ± 2.65 (adjusted mean ± SE) 95% CI [4.44~6.01] versus control group 7.43 ± 2.80 [6.59~8.26], *P* = 0.0001 (Table 3). Significant differences in total M-JOA score were also evident during the follow-up period (*P* = 0.0006).

#### 3.2.2. Knee Circumference

Reductions in mean knee circumference at months 1 and 7 compared with control group were observed, and they were significant. Significant differences presented between the groups (*P* < 0.05) were shown in Table 4.

### 3.3. Safety

No adverse events were reported in the 90 participants.

## 4. Discussions

In this observational comparative effectiveness study, patients with KOA who presented heat-sensitive moxibustion sensation significantly reduced pain and possessed better function after one month than the ones who received conventional local superficial heat sensation, according to the total GPCRND-KOA score. Both of the groups substantially improved during the observation period. After 7 months, exploratory analysis indicated that the differences between the two groups were still significant. Significant differences were also evident for knee circumference.

The design of the study (observational and multicenter setting) allows evaluation of a therapy's comparative effectiveness considering the acupuncture point's own reactions and the Deqi sensation. Both the evaluation of the results and the statistical analysis were carried out in a blind fashion to improve the objectivity and validity of the study outcomes.

The aim of this study was to compare the Deqi effect and non-Deqi effect stimulated by suspended moxibustion and to confirm that heat-sensitive moxibustion sensation simulated by moxibustion is a sign of the Deqi. Thus, Deqi sensation occurrence preferences were chosen to take it into account, and it made randomization not possible. The observational design resulted in relevant baseline differences of the two groups. In the trial group GPCRND-KOA appeared higher compared with the conventional group. In the previous treatment, there were obvious differences between the two groups. To take baseline differences into account, we adjusted our analyses of these factors. However, it is possible that other unknown and unmeasured factors might have influenced the results. Therefore, the nonrandomized design is a clear limitation of our study considering the internal validity of our results.

In this study, we investigated the relationship between the Deqi sensation and therapeutic effect according to moxibustion stimulation. Previous studies manifested that the Deqi (heat-sensitive moxibustion sensation) was elicited in 70% of the moxibustion procedures of patients [21]. The frequency and intensity of individual sensations were significantly higher in KOA. Among the sensations typically associated with the Deqi, penetrating heat, expanding heat, and transmitting heat were most common [22]. Being consistent with their prominent roles in TCM, bilateral Xi Yan (EX-LE5) and He Ding (EX-LE2) showed the most prominent sensations [13, 21]. 

In terms of the Deqi sensation of acupuncture needle, it has been demonstrated that most of the Deqi sensations are conveyed by different nerve fiber systems. Aching, soreness, distension, heaviness, warmth, and dull pain are conveyed by the slower conducting A*δ* and C fibers, whereas numbness is conveyed by the faster conducting A*β* fibers [23, 24]. However, there is lack of experimental data to indicate the basic substances that contribute to the Deqi sensation of moxibustion. Further research is required to discover the underlying mechanisms of the Deqi in moxibustion.

Both the total GPCRND-KOA score and reduction in knee circumference were evident in the Deqi sensation of heat-sensitive phenomenon and conventional local superficial heat sensation by applying suspended moxibustion. What is more important is that our trial result conforms to the theory of TCM, “the arrival of meridian Qi ensures the therapeutic effects.” The effectiveness of the Deqi sensation group might be more superior than the non-Deqi sensation one in the treatment of KOA. In a word, this study is aimed at providing scientific evidence for the Deqi sensation of moxibustion and at showing that heat-sensitive moxibustion sensation is essential to achieve the preferable treatment effects for KOA.

## Figures and Tables

**Table 1:** List of GPCRND-KOA.

Item	Grade/classification	Score
Pain or discomfort in night when lying in bed	No	0
	Pain in activity or some position	1
	Pain in nonactivity	2

Morning stiffness or pain worse when getting out of bed	No	0
	<30 minutes	1
	≥30 minutes	2

Pain or discomfort in walk	No	0
	After walking in some distance	1
	Pain at beginning of walk or worse	2

Arise from seat	Independent	0
	Need assistance	1

The maximum walk distance (accompanied with pain)	Unrestricted	0
	Restricted, >1 km	1
	300 m*∼*1 km	2
	<300 m	3

Daily activities	Board standard airstairs	
	Independent	0
	Difficulty	1
	Unable	2
	Step down standard airstairs	
	Independent	0
	Difficulty	1
	Unable	2
	Squat or bend knees	
	Independent	0
	Difficulty	1
	Unable	2
	Walk over rough terrain	
	Independent	0
	Difficulty	1
	Unable	2

**Table 2:** Baseline characteristics of study patients.

Items	Trial group	Control group	*P* value
Age, mean (SD), years	56.13 (7.55)	59.34 (7.21)	0.04
Sex *n* (%)			0.0002
Female	12 (26.67)	32 (71.11)	
Male	33 (73.33)	13 (28.89)	
Duration of knee pain *n* (%)			0.67
<5 years	33 (73.33)	30 (66.67)	
5–10 years	9 (20.00)	11 (24.44)	
>10 years	3 (6.67)	4 (8.89)	
BMI, mean (SD), kg/m′	22.12 (3.12)	24.22 (3.30)	0.002
GPCRND-KOA grade *n* (%)			0.51
Severe	31 (68.89)	28 (62.22)	
Moderate	14 (31.11)	17 (37.78)	
Knee circumference, mean (SD), cm	40.26 (3.31)	42.21 (3.25)	0.005
GPCRND-KOA score, mean (SD)	13.45 (3.28)	11.12 (3.13)	0.0006
Previous treatment (past half year, %)			0.041
Pharmaceutical intervention	31 (68.89)	18 (40.00)	
Physiotherapy	11 (24.44)	20 (44.44)	
Previous acupuncture treatment	3 (6.67)	5 (11.11)	

BMI: Body Mass Index; GPCRND-KOA: guiding principle of clinical research on new drugs in the treatment of KOA score; SD: standard deviation; KOA: knee osteoarthritis.

**Table 3:** Comparison of GPCRND-KOA scores.

Variable	Month 1		Month 7	
	Mean	95% CI	Mean	95% CI
Trial group	5.23	4.44*∼*6.01	4.78	4.37*∼*5.18
Control group	7.43	6.59*∼*8.26	6.11	5.45*∼*6.76
*P* value	0.0001		0.0006	

*Adjusted means or proportions and confidence intervals (CI) from multilevel models (ANCOVA or GEE) with fixed effects. All data are intended to treat. Both groups *n* = 45. SD: standard deviation; GPCRND-KOA: guiding principle of clinical research on new drugs in the treatment of KOA score; KOA: knee osteoarthritis.

**Table 4:** Comparison of knee circumference.

Variable	Month 1		Month 7	
	Mean	95% CI	Mean	95% CI
Trial group	38.32	37.07*∼*39.56	37.22	36.24*∼*38.19
Control group	40.30	39.95*∼*41.64	39.10	38.39*∼*40.19
*P* value	0.03		0.007	

*Adjusted means or proportions and confidence intervals (CI) from multilevel models (ANCOVA or GEE) with fixed effects. All data are intended to treat. Both groups *n* = 45. SD: standard deviation; GPCRND-KOA: guiding principle of clinical research on new drugs in the treatment of KOA score; KOA: knee osteoarthritis.
